# Identification of nine novel loci related to hematological traits in a Japanese population

**DOI:** 10.1152/physiolgenomics.00088.2017

**Published:** 2018-06-29

**Authors:** Yoshiki Yasukochi, Jun Sakuma, Ichiro Takeuchi, Kimihiko Kato, Mitsutoshi Oguri, Tetsuo Fujimaki, Hideki Horibe, Yoshiji Yamada

**Affiliations:** ^1^Department of Human Functional Genomics, Advanced Science Research Promotion Center, Mie University, Tsu, Mie, Japan; ^2^CREST, Japan Science and Technology Agency, Kawaguchi, Saitama, Japan; ^3^Computer Science Department, College of Information Science, University of Tsukuba, Tsukuba, Ibaraki, Japan; ^4^RIKEN Center for Advanced Intelligence Project, Tokyo, Japan; ^5^Department of Computer Science, Nagoya Institute of Technology, Nagoya, Aichi, Japan; ^6^Department of Internal Medicine, Meitoh Hospital, Nagoya, Aichi, Japan; ^7^Department of Cardiology, Kasugai Municipal Hospital, Kasugai, Aichi, Japan; ^8^Department of Cardiovascular Medicine, Inabe General Hospital, Inabe, Mie, Japan; ^9^Department of Cardiovascular Medicine, Gifu Prefectural Tajimi Hospital, Tajimi, Gifu, Japan

**Keywords:** exome-wide association study, generalized estimating equation, hematological trait, linkage disequilibrium, longitudinal data

## Abstract

Recent genome-wide association studies have identified various genetic variants associated with hematological traits. Although it is possible that quantitative data of hematological traits are varied among the years examined, conventional genome-wide association studies have been conducted in a cross-sectional manner that measures traits at a single point in time. To address this issue, we have traced blood profiles in 4,884 Japanese individuals who underwent annual health check-ups for several years. In the present study, longitudinal exome-wide association studies were conducted to identify genetic variants related to 13 hematological phenotypes. The generalized estimating equation model showed that a total of 67 single nucleotide polymorphisms (SNPs) were significantly [false discovery rate (FDR) of <0.01] associated with hematological phenotypes. Of the 67 SNPs, nine SNPs were identified as novel hematological markers: rs4686683 of *SENP2* for red blood cell count (FDR = 0.008, *P* = 5.5 × 10^−6^); rs3917688 of *SELP* for mean corpuscular volume (FDR = 0.005, *P* = 2.4 × 10^−6^); rs3133745 of *C8orf37-AS1* for white blood cell count (FDR = 0.003, *P* = 1.3 × 10^−6^); rs13121954 at 4q31.2 for basophil count (FDR = 0.007, *P* = 3.1 × 10^−5^); rs7584099 at 2q22.3 (FDR = 2.6 × 10^−5^, *P* = 8.8 × 10^−8^), rs1579219 of *HCG17* (FDR = 0.003, *P* = 2.0 × 10^−5^), and rs10757049 of *DENND4C* (FDR = 0.008, *P* = 5.6 × 10^−5^) for eosinophil count; rs12338 of *CTSB* for neutrophil count (FDR = 0.007, *P* = 2.9 × 10^−5^); and rs395967 of *OSMR-AS1* for monocyte count (FDR = 0.008, *P* = 3.2 × 10^−5^).

## INTRODUCTION

Hematological traits are important for determining the health status or diagnosing diseases. Blood cells are classified into three major blood-cell lineages: red blood cells (RBCs), white blood cells (WBCs), and platelets. Recent genome-wide association studies (GWASs) have identified various genetic variants associated with hematological traits in diverse ethnic groups. Mousas et al. (2017) (29) examined relations of 137,086 rare coding or splice genetic variants [minor allele frequency (MAF) of <0.01] to 15 hematological traits in 308,572 subjects with European ancestry. They showed that 56 variants were associated with hematological phenotypes and identified a novel association of rs145535174 in *PLG* with platelet counts (29). Okada and Kamatani (2012) (31) systematically reviewed genetic association studies for hematological traits in several ethnic populations and reported that >100 genetic loci had been identified to affect the hematological phenotypes. Despite a number of associated loci, additional novel relations between genetic variants and hematological quantitative traits have been found to date (2, 16, 20, 35, 37). While some loci related to hematological traits are shared among ethnic populations, interethnic differences in the associated loci have been observed (15, 20, 23, 35). In Japan, however, there have been few GWASs for multiple hematological traits (15, 30). Hence, it is possible that genetic variants associated with hematological dynamics in Japanese individuals remain to be identified definitively.

Most conventional GWASs have been conducted in a cross-sectional manner that measured traits at a single point in time. Since the number of blood cells in Japanese adults is known to decrease with age (18), it is possible that quantitative data of hematological traits in an individual varied greatly among the years examined. However, the cross-sectional GWASs did not consider this possibility. Given that a longitudinal study examines longitudinal hematological data, this analysis increases the statistical power to detect the association. Therefore, hematological profiles in 4,884 Japanese individuals who underwent annual health check-ups for several years have been traced. We have now performed longitudinal exome-wide association studies to explore novel loci associated with 13 hematological traits: RBC count; hemoglobin (Hb); hematocrit (Ht); mean corpuscular volume (MCV); mean corpuscular hemoglobin (MCH); mean corpuscular hemoglobin concentration (MCHC); platelet count; WBC count; and neutrophil, basophil, eosinophil, lymphocyte, and monocyte counts.

## MATERIALS AND METHODS

### 

#### Compliance with ethical standards.

The study protocol complied with the Declaration of Helsinki and was approved by the Committees on the Ethics of Human Research of Mie University Graduate School of Medicine and Inabe General Hospital. Written informed consent was obtained from all subjects before enrollment in the study.

#### Study subjects.

Study subjects comprised a total of 4,884 community-dwelling individuals (2,792 men and 2,092 women) who visited the Health Care Center of Inabe General Hospital (Inabe, Mie, Japan) for annual health check-ups from April 2003 to March 2014. All participants had each undergone 1–11 medical examinations, and the average follow-up period, which was equivalent to the average number of measurements, was 5 yr. We refer to this cohort as the “Inabe cohort.” Methods for the collection and storage of medical examination data and genomic DNA samples have been described previously (46). Patients with active cancers or pregnant women were not included in the study.

To assess genetic association, we used repeated measurements of 13 hematological traits in 4,884 Inabe subjects. The 13 traits consist of *1*) RBC count, the number of RBCs in peripheral venous blood (10^12^/l); *2*) Hb, the concentration of hemoglobin in the blood (g/dl); *3*) Ht, the volume percentage of RBCs in the blood (%); *4*) MCV, the average volume of RBC that is expressed in femtoliters (fl), = Ht × 10/[RBC (10^12^/l)]; *5*) MCH, the average mass of Hb per RBC in the sample (pg), = Hb × 10/[RBC (10^12^/l)]; *6*) MCHC, the average concentration of Hb per RBC contained in the sample (g/dl), = Hb × 100/Ht; *7*) platelet count, the number of platelets in the blood (10^9^/l); *8*) WBC count, the number of WBCs in the blood (10^9^/l); and *9–13*) five WBC subtypes (neutrophils, basophils, eosinophils, lymphocytes, and monocytes), the percentage of each subtype in WBCs in the sample (%). We used an automated hematology analyzer LH-780 (Beckman Coulter, Brea, CA) to measure hematological traits of participants. The measurements were performed with a single equipment in the single clinical laboratory of the hospital.

**Fig. 1.**
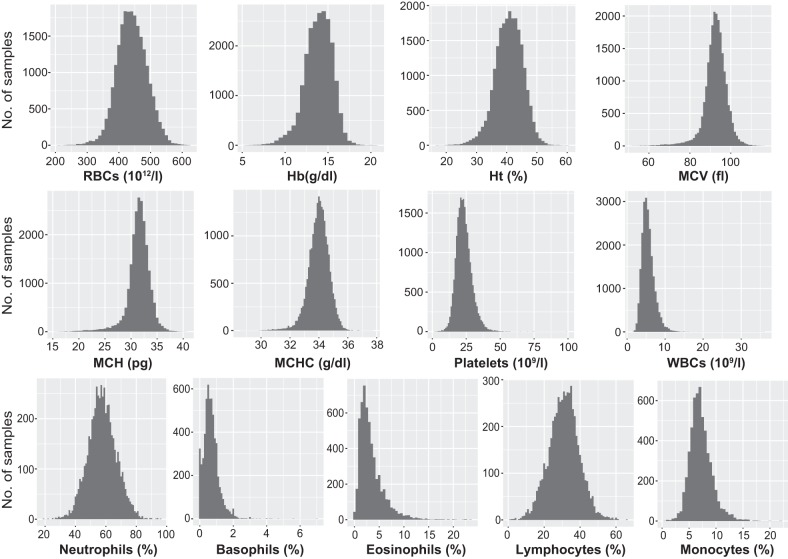
Count distributions for longitudinal data of 13 hematological traits examined in the Inabe cohort (4,884 individuals). RBC, red blood cell count; Hb, hemoglobin; Ht, hematocrit; MCV, mean corpuscular volume; MCH, mean corpuscular hemoglobin; MCHC, mean corpuscular hemoglobin concentration; WBC, white blood cell count.

The distributions of longitudinal data in the 13 hematological traits are shown in Fig. 1. In the longitudinal exome-wide association studies for RBCs, Hb, and MCH, a total of 4,884 subjects (2,792 men and 2,092 women) were examined (21,828–22,146 examinations). A total of 4,883 subjects (2,791 men and 2,092 women) were examined in the exome-wide association studies for Ht, MCV, and MCHC (21,828–22,133 examinations). Furthermore, 4,826 subjects (2,757 men and 2,069 women) and 4,833 subjects (2,762 men and 2,071 women) were examined in the exome-wide association studies for platelets (21,865 examinations) and WBCs (21,936 examinations), respectively. In the exome-wide association studies for the five WBC subtypes (neutrophils, basophils, eosinophils, lymphocytes, and monocytes), 1,073 subjects (5,591 examinations) for monocytes and 1,072 subjects (5,589–5,590 examinations) for the other subtypes were examined.

#### Longitudinal exome-wide association study.

Longitudinal exome-wide association studies for the Inabe subjects were conducted, based on ~244,000 genetic variants and longitudinal data of the 13 hematological traits. Genotyping for the subjects was performed with the Infinium HumanExome-12 ver. 1.2 BeadChip and Infinium Exome-24 ver. 1.0 BeadChip (Illumina, San Diego, CA). These arrays included putative functional exonic variants selected from >12,000 individual exome and whole-genome sequences across diverse ethnic populations, including European, African, Chinese, and Hispanic individuals (11). Approximately 3.6% of all the genetic variants were excluded from further analyses because one of the exome arrays did not contain them. We performed quality controls and discarded the following genotyped sites: *1*) monomorphic sites among Inabe subjects, *2*) variants with MAF of <0.05, *3*) variants whose genotype distribution significantly (*P* < 0.001) deviated from the Hardy-Weinberg equilibrium in the subjects. Analysis of the association of genetic variants on sex chromosomes with phenotypes is complicated because of the difference in the copy number between men and women and of X-inactivation in women. Analysis of the association of genetic variants on mitochondrial DNA with phenotypes is also complicated because of the existence of heteroplasmy. We thus discarded genetic variants located on sex chromosomes and mitochondrial DNA. Among the Inabe subjects, no individual was identified as population outliers by principal component analysis of genetic variants using the EIGENSTRAT method (33) via JMP Genomics version 6.0 (SAS Institute, Cary, NC) to detect population stratification on an exome-wide scale. Consequently, a total of 24,642 single nucleotide polymorphisms (SNPs) among 4,884 samples passed quality control.

**Fig. 2.**
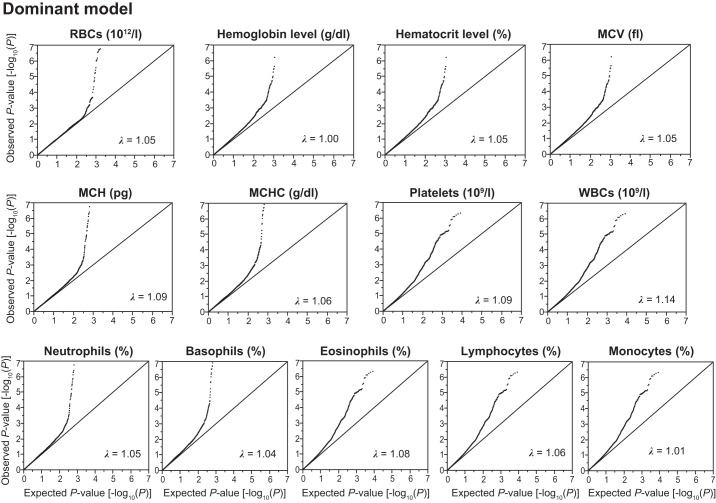
Quantile-quantile plots for *P* values in the longitudinal exome-wide association studies for 13 hematological traits in the dominant model. The observed *P* values (*y*-axis) were compared with the expected *P* values (*x*-axis) under the null hypothesis, with the values being plotted as –log_10_(*P*). RBC, red blood cell count; MCV, mean corpuscular volume; MCH, mean corpuscular hemoglobin; MCHC, mean corpuscular hemoglobin concentration; WBC, white blood cell count; λ represents the genomic inflation factor.

**Fig. 3.**
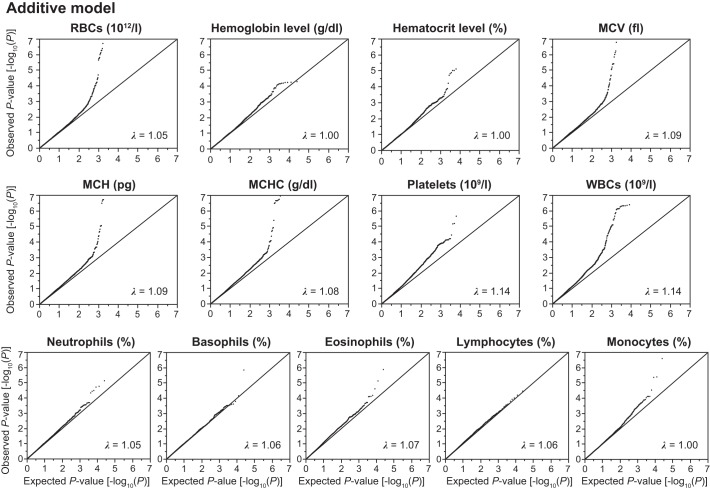
Quantile-quantile plots for *P* values in the longitudinal exome-wide association studies for 13 hematological traits in the additive model. The observed *P* values (*y*-axis) were compared with the expected *P* values (*x*-axis) under the null hypothesis, with the values being plotted as –log_10_(*P*). RBC, red blood cell count; MCV, mean corpuscular volume; MCH, mean corpuscular hemoglobin; MCHC, mean corpuscular hemoglobin concentration; WBC, white blood cell count. λ represents the genomic inflation factor.

**Fig. 4.**
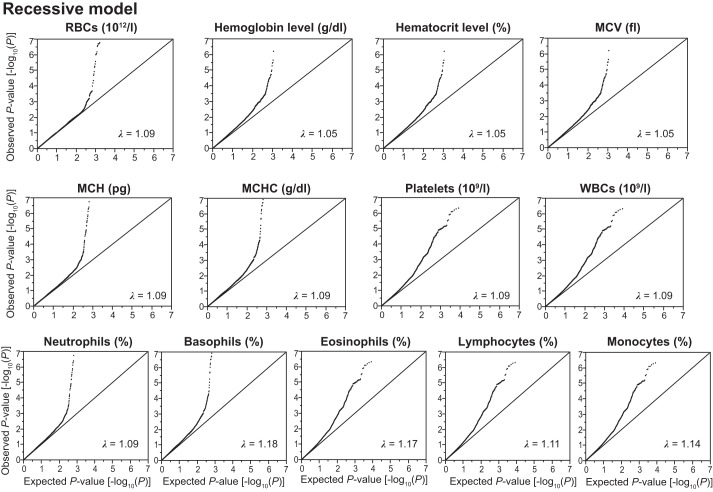
Quantile-quantile plots for *P* values in the longitudinal exome-wide association studies for 13 hematological traits in the recessive model. The observed *P* values (*y*-axis) were compared with the expected *P* values (*x*-axis) under the null hypothesis, with the values being plotted as –log_10_(*P*). RBC, red blood cell count; MCV, mean corpuscular volume; MCH, mean corpuscular hemoglobin; MCHC, mean corpuscular hemoglobin concentration; WBC, white blood cell count. λ represents the genomic inflation factor.

Given that effects of SNPs on phenotypes may differ among three inheritance models, we examined the association of SNPs with hematological phenotypes in three inheritance models. We thus converted the genotyping data of 4,884 Inabe subjects into numeric data with dominant, additive, and recessive models. The dominant and recessive models were defined as “AA (0) vs. AB + BB (1)” and “AA + AB (0) vs. BB (1)” (A, major allele; B, minor allele), respectively, whereas the additive model was defined as “AA (0), AB (1), BB (2).” Quantile-quantile plots for *P* values of allele frequencies in the exome-wide association studies are shown in Figs. 2–4. The genomic inflation factor (λ) of *P* values was 1.00–1.14 for the 13 hematological traits in the dominant and additive models (Figs. 2 and 3). In the recessive model, the λ was 1.05–1.18 (Fig. 4). The rearrangement of Inabe longitudinal data was conducted in the R software version 3.32 (34) via RStudio version 1.0.136 (36) and by the use of a Perl script. We surveyed the linkage disequilibrium (LD) between the focal SNPs and other genetic variants that have been shown to be associated with hematological traits in previous studies through LDlink web-based tools (https://ldlink.nci.nih.gov) (25).

#### Statistical analyses.

We examined relations between SNPs and repeated measurements of hematological traits by the generalized estimating equation (GEE) model (14, 21) with adjustments for age and sex done with the R package “geepack” (13). The “waves” argument was used to specify the ordering of repeated measurements within individuals, because this argument is required to recognize clusters of each individual. Given that many SNPs in exome arrays are in LD, effects of SNPs on hematological traits are not independent. Therefore, we calculated the false discovery rate (FDR) by the Benjamin and Hochberg method (4) to compensate for multiple comparison of genotypes with the hematological parameters. An FDR of <0.01 was considered for statistical significance of association. Sitlani et al. (2015) (40) reported that a small effective sample size can increase the chances of generating type I errors. They recommended the use of “approxdf” for an effective sample size: approxdf = 2 × MAF × Nindep, where Nindep is the sum of the estimated number of independent observations per person, and an approxdf of ≥10 could reduce type I errors. Therefore, we estimated the approxdf for each candidate SNP by the R package “bosswithdf” (40, 44). To solve the issue of association with small effective sample sizes, we set to strict approxdf threshold, and discarded SNPs with approxdf of ≤30. The GEE model with the R package geepack was used to examine the association of SNPs with repeated measurements. To consider the association of SNPs with time-dependent phenotypic changes, we also calculated the *P* value with GEE using a *t* reference distribution with Satterthwaite estimates of degrees of freedom by the bosswithdf package, and incorporated a SNP × time interaction term into this model for time dependent analyses. The GEE with the *t* reference distribution was implemented using the longitudinal hematological data of subjects in the last five years (equivalent to the mean follow-up period).

#### Prediction of functional association for candidate loci.

Gene-gene functional interactions were predicted using GeneMANIA Cytoscape plugin (27, 28, 45) via Cytoscape version 3.4.0 (38) software. Relations of candidate SNPs to hematological phenotypes reported by previous studies were investigated by GRASP (Genome-Wide Repository of Associations Between SNPs and Phenotypes, https://grasp.nhlbi.nih.gov/Overview.aspx) (19) and GWAS Catalogue (https://www.ebi.ac.uk/gwas/) (24) databases.

## RESULTS

### 

#### Characteristics of subjects.

The characteristics of 4,884 Inabe subjects with respect to the longitudinal data of 13 hematological traits are shown in Table 1. The mean ages of men and women were 52.0 yr (range: 19–89 yr) and 52.2 yr (range: 18–91 yr of age), respectively.

**Table 1.** Longitudinal characteristics of 4,884 Inabe subjects

Characteristic	Subjects, *n*	Examinations, *n*	Means ± SD	Range
Age, yr	4,884	22,146	52.1 ± 12.8	18–91
RBCs, 10^12^/l	4,884	22,145	4.4 ± 0.5	2.1–6.2
Hb, g/dl	4,884	22,146	13.9 ± 1.6	5.5–20.6
Ht, %	4,883	22,133	40.8 ± 4.6	17.4–59.9
MCV, fl	4,883	21,828	92.5 ± 5.6	52.1–115.9
MCH, pg	4,884	21,828	31.5 ± 2.2	15.3–40.5
MCHC, g/dl	4,883	21,828	34.0 ± 0.7	28.6–37.7
Platelets, 10^9^/l	4,826	21,865	234 ± 58	17–990
WBCs, 10^9^/l	4,833	21,936	5.5 ± 1.7	1.5–36.1
Neutrophils, %	1,072	5,590	57.6 ± 9.3	18.5–96.4
Basophils, %	1,072	5,590	0.7 ± 0.5	0–7.0
Eosinophils, %	1,072	5,589	3.4 ± 2.5	0–23.5
Lymphocytes, %	1,072	5,590	31.1 ± 8.4	1.1–66.2
Monocytes, %)	1,073	5,591	7.2 ± 2.1	0.7–22.0

The number of subjects is based on data examined in the latest year. Examination values indicate the numbers of measurements taken. Quantitative data are means ± SD. RBC, red blood cell count; Hb, hemoglobin; Ht, hematocrit; MCV, mean corpuscular volume; MCH, mean corpuscular hemoglobin; MCHC, mean corpuscular hemoglobin concentration; WBC, white blood cell count.

#### Longitudinal exome-wide association studies for RBC traits and platelets.

We assessed significant association between 13 hematological traits and SNPs that passed quality control in the three inheritance models with the GEE model. Candidate SNPs detected in all the inheritance models are shown in Supplemental Table S1. (The online version of this article contains supplemental material.) Our longitudinal exome-wide association studies showed that a total of 67 SNPs were significantly (FDR <0.01) associated with hematological traits examined (Supplemental Table S2). Allele frequencies of these candidate SNPs are shown in Supplemental Table S3.

In longitudinal exome-wide association studies for six RBC traits, the GEE model showed 26 SNPs associated with RBCs, three SNPs with Ht, 25 SNPs with MCV, 17 SNPs with MCH, and 14 SNPs with MCHC, whereas there were no SNPs associated with Hb (Supplemental Tables S1 and S2). According to GRASP and GWAS Catalogue databases, 10 SNPs have not been reported by previous studies. However, previously known SNPs were located around the eight of 10 candidate SNPs, and these SNPs (four SNPs around *ABO* at the chromosomal region 9q34.2 and the different set of four SNPs around *ALDH2* at 12q24.1) were possibly in LD with the known SNPs related to the RBC traits examined. Thus, we did not consider candidate SNPs that were possibly correlated with the known associated SNPs as novel hematological markers. Consequently, we newly identified two SNPs related to RBC traits: rs4686683 of *SENP2* for RBCs (FDR = 0.008, *P* = 5.5 × 10^−6^, approxdf = 223) and rs3917688 of *SELP* for MCV (FDR = 0.005, *P* = 2.4 × 10^−6^, approxdf = 45) (Supplemental Table S2). Mean quantitative values of each hematological measurement in individuals with different genotypes at the novel candidate SNPs are shown in Supplemental Table S4.

In the longitudinal exome-wide association studies for platelets, two SNPs were significantly related to platelet count (Supplemental Tables S1 and S2). However, these SNPs have been shown to be associated with hematological phenotypes, according to GRASP and GWAS Catalogue databases. Therefore, there were no novel genetic determinants for platelets in our longitudinal exome-wide association studies.

#### Longitudinal exome-wide association study for WBC traits.

We performed longitudinal exome-wide association studies for six WBC traits and found 19 SNPs associated with WBCs, one SNP with basophils, three SNPs with eosinophils, one SNP with neutrophils, and two SNPs with monocytes (Supplemental Table S2). No significant association with lymphocytes was observed in the present study. Of the 26 SNPs, the association of 24 has not been reported by previous studies according to the two databases. However, 17 were possibly in LD with previously reported SNPs related to WBC traits.

**Fig. 5.**
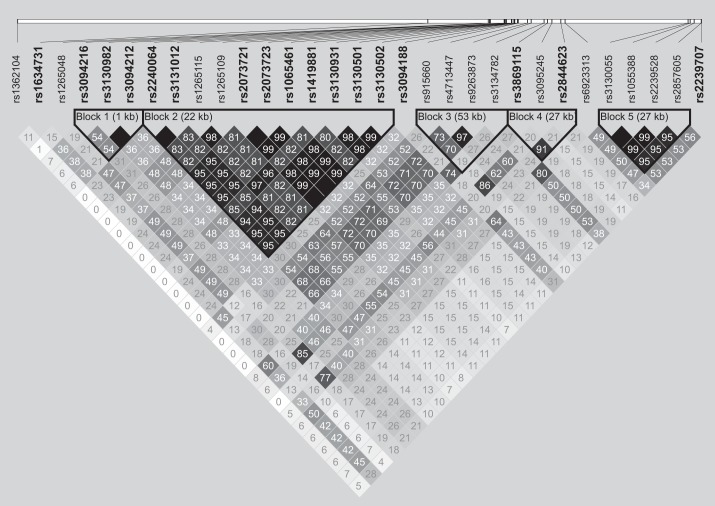
Linkage disequilibrium of biallelic sites in a ~1.4 Mb genomic region at 6p21.3–p22.1 based on single nucleotide polymorphism (SNP) data from 4,884 Inabe residents. The SNPs with the minor allele frequency of <0.01 were removed from the analysis. The white blood cell-associated SNPs are shown in boldface.

Of the 17 SNPs, the GEE model showed significant relations of 15 SNPs across a ~569 kb genomic region at 6p21.33 (in the *HLA* region) to WBCs (Supplemental Tables S1 and S2). Although there have been no reports of association between these SNPs and hematological traits examined, previous studies have reported association of SNPs in the *HLA* region with WBCs (15, 30, 31). On the basis of SNPs in the Inabe cohort, we generated an LD plot in a ~1.4 Mb genomic region at 6p21.3–p22.1 with the Haploview program version 4.2 (3) (Fig. 5). The plot showed moderate or strong correlations between pairs of SNPs examined. Since long-range haplotypes in the *HLA* region have been maintained under natural selection (12, 32), it is difficult to distinguish which SNP is responsible for hematological dynamics in individuals. Therefore, we did not consider the 15 SNPs at 6p21.33 as novel candidate hematological markers, although it is possible that any of these SNPs are actually associated with hematological traits.

The GEE model showed novel relations of rs56030650 in *GSDMA* and rs10107630 in *CCDC26* to WBCs and monocytes, respectively (Supplemental Table S2). However, other SNPs in these genes (rs3859192 in *GSDMA* and rs10956483 in *CCDC26*) have been shown to be related to the corresponding WBC traits (6, 30). LDpair, an LDlink application, indicated that rs56030650 and rs10107630 were in LD with rs3859192 (*r^2^* = 0.98, Dʹ = 1.00) and rs10956483 (*r^2^* = 0.88, Dʹ = 0.94), respectively, in JPT (Japanese in Tokyo, Japan) from the 1000 Genomes Project database (http://www.internationalgenome.org) (43). Hence, we did not consider the two candidate SNPs as novel hematological markers.

We thus identified the following seven SNPs as novel genetic determinants for WBC traits: rs3133745 of *C8orf37-AS1* for WBCs (FDR = 0.003, *P* = 1.3 × 10^−6^, approxdf = 163), rs13121954 at the chromosomal region 4q31.2 for basophils (FDR = 0.007, *P* = 3.1 × 10^−5^, approxdf = 47), rs7584099 at 2q22.3 for eosinophils (FDR = 2.6 × 10^−5^, *P* = 8.8 × 10^−8^, approxdf = 33), rs1579219 of *HCG17* for eosinophils (FDR = 0.003, *P* = 2.0 × 10^−5^, approxdf = 37), rs10757049 of *DENND4C* for eosinophils (FDR = 0.008, *P* = 5.6 × 10^−5^, approxdf = 40), rs12338 of *CTSB* for neutrophils (FDR = 0.007, *P* = 2.9 × 10^−5^, approxdf = 49), and rs395967 of *OSMR-AS1* for monocytes (FDR = 0.008, *P* = 3.2 × 10^−5^, approxdf = 49).

#### Additional exome-wide association studies.

To examine the association of the nine candidate SNPs with the time-varying phenotypes, we applied the GEE model with a *t* reference distribution including a SNP × time interaction term (see materials and methods). This analysis showed that four of nine candidate SNPs were associated (*P* < 0.05) with the longitudinal changes of hematological trait (rs4686683 of *SENP2* for RBCs, rs13121954 at 4q31.2 for basophils, rs12338 of *CTSB* for neutrophils, and rs1579219 of *HCG17* for eosinophils).

In the present study, an FDR of <0.01 is considered statistically significant. If Bonferroni’s correction [a *P* value of <2.0 × 10^−6^ (0.05/24,642 SNPs)] is applied, relations of rs7584099 at 2q22.3 (*P* = 8.8 × 10^−8^) and rs3133745 of *C8orf37-AS1* (*P* = 1.3 × 10^−6^) to eosinophils and WBCs, respectively, were significant. Since the remaining seven SNPs did not reach the threshold of *P* value with Bonferroni’s correction, the association of the seven SNPs with hematological phenotypes should be carefully interpreted, and replication studies are required to verify the association.

To detect the association of rare population-specific variants, we conducted longitudinal exome-wide association studies for hematological traits, using 41,442 genetic variants including low-frequency and rare variants (0.1 ≤ MAF ≤ 5%). Even though a large number of genetic variants were significantly (FDR <0.01) associated with hematological phenotypes, all of these variants with a MAF of <5% were filtered out by an approxdf threshold of >30.

#### Association of newly identified and previously reported SNPs with hematological phenotypes.

We examined 67 SNPs related to hematological phenotypes in the present study with information of *P* values in three Blood-Cell Consortium (BCX) studies (5, 9, 41) from the website Laboratory of Guillaume Lettre (http://www.mhi-humangenetics.org). All the three, 17, two, and one SNPs associated with Ht, MCH, platelets, and basophils, respectively, in our longitudinal exome-wide association studies were also related (*P* < 0.05) to their phenotypes in the previous studies (Supplemental Table S5). There were no SNPs associated with eosinophils in the previous studies, although three SNPs were related to eosinophils in our longitudinal exome-wide association studies. The association of many SNPs was observed in the previous studies: nine of 14 SNPs associated with MCHC, 19 of 23 SNPs associated with MCV, one of two SNPs associated with monocytes, 22 of 25 SNPs associated with RBC counts, and 13 of 18 SNPs associated with WBC counts. Of the nine SNPs that were identified as novel hematological markers in the present study, rs13121954 were associated with basophils in both previous and present studies. In the BCX studies, no information on *P* values for rs12338 was available, and six SNPs were related to different hematological phenotypes from the present study: rs3917688 associated with basophils and platelets, rs395967 with red blood cell distribution width (RDW), rs1579219 with neutrophils, rs3133745 with RDW and neutrophils, and rs10757049 with MCH and RBC. Two SNPs (rs7584099 and rs4686683) were not associated with any hematological phenotypes in the previous studies.

We compared the effect direction and allele frequency of effect allele in subjects with East Asian and European ancestries (Supplemental Table S6). In the comparison of effect direction of 63 SNPs between Inabe subjects (East Asian ancestry) and Europeans in BCX studies, the consistency of the direction in 20–48 SNPs was observed: 34 SNPs for basophils, 36 for eosinophils, 48 for Ht, 45 for Hb, 28 for lymphocytes, 44 for MCH, 39 for MCHC, 43 for MCV, 20 for monocytes, 29 for neutrophils, 47 platelets, 41 for RBC counts, and 40 for WBC counts. Of the eight newly identified SNPs (no information of *P* values for rs12338), three (rs7584099, rs4686683, and rs1579219) showed the same direction of association. Although frequencies of effect alleles were different between the subjects with East Asian and European ancestries, the effect direction was the same [e.g., the effect direction of association in rs4686683 was same between the two groups although the frequencies of effect allele were different between Inabe subjects (0.399) and Europeans (0.613)].

We also examined 494 variants (including variants with an MAF of <0.05) identified in the BCX studies (*P* < 2.0 × 10^−7^, significance level in the studies), using data of our longitudinal exome-wide association studies (Supplemental Table S7). Of the 494 variants, 329 were associated (*P* < 0.05) with at least one of hematological phenotypes examined in the additive model. Of these variants examined, moreover, 41–92 SNPs were associated with each hematological trait examined: 90 SNPs for RBC counts, 86 for Hb, 83 for Ht, 83 for MCV, 91 for MCH, 89 for MCHC, 92 for platelets, 79 for WBC counts, 47 for neutrophils, 38 for basophils, 42 for eosinophils, 41 for lymphocytes, and 55 for monocytes.

We additionally examined the association of 67 SNPs with hematological traits examined in our exome-wide association studies, using the data set of Astle et al. (2016) (1) in the GRASP database (Supplemental Table S8). The information on *P* values for 40 SNPs including seven newly identified SNPs is available in the data set. All SNPs associated with Ht (3 SNPs), MCH (16), MCHC (8), platelets (2), and eosinophils (1) in our longitudinal exome-wide association studies were also related (*P* < 0.05) to the corresponding phenotypes in the previous studies. The rs13121954 was not associated with basophils. The number of difference of SNPs associated with the following phenotypes was only one between present and previous studies: MCV (18 SNPs), monocytes (1), RBC counts (19), and WBC counts (6) in the previous study. Of the seven newly identified SNPs, rs7584099 and rs4686683 were associated with eosinophils and RBCs, respectively, in both previous and present studies.

In the comparison of effect direction of 40 SNPs between the Inabe subjects and Europeans in Astle et al. (2016) (1), the consistency of the direction in 12–35 SNPs was observed: 24 SNPs for basophils, 26 for eosinophils, 33 for Ht, 35 for Hb, 16 for lymphocytes, 31 for MCH, 29 for MCHC, 31 for MCV, 12 for monocytes, 20 for neutrophils, 32 platelets, 32 for RBC counts, and 31 for WBC counts (Supplemental Table S9). Of the seven newly identified SNPs (no information on *P* values for rs12338 and rs1579219), four (rs3917688, rs4686683, rs395967, and rs3133745) showed the same direction of association.

We also examined 80 variants identified (*P* < 2.0 × 10^−7^) in Astle et al. (2016) (1), by the use of data in our longitudinal exome-wide association studies (Supplemental Table S10) and observed that 50 variants were related (*P* < 0.05) to at least one of hematological phenotypes in the additive model. Of the 80 variants examined, 7–13 SNPs were associated with each hematological trait examined: 11 SNPs for RBC counts, seven for Hb, nine for Ht, 11 for MCV, 10 for MCH, 11 for MCHC, 13 for platelets, seven for WBC counts, nine for neutrophils, eight for basophils, eight for eosinophils, eight for lymphocytes, and nine for monocytes.

#### Gene interaction network analysis.

**Fig. 6.**
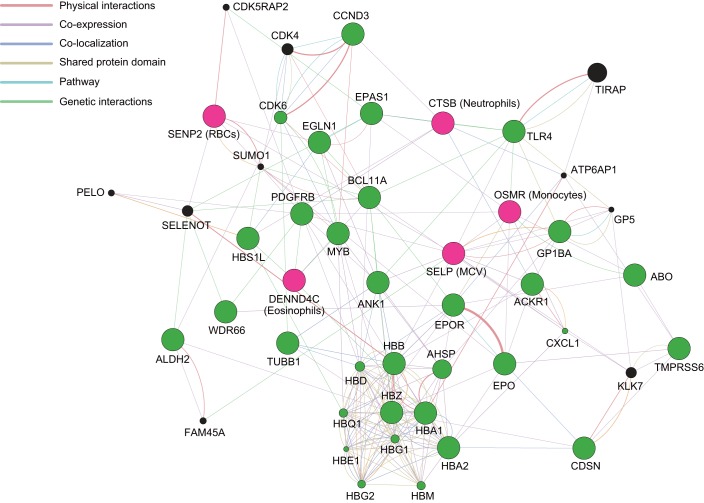
Diagram of GeneMANIA network analysis for newly identified (pink circles) and previously reported (green circles) genes related to hematological dynamics. The hematological traits associated with newly identified SNPs are shown in parentheses.

To explore interactive functional association among newly identified and previously known genes related to hematological dynamics, we preliminary performed a GeneMANIA network analysis (Fig. 6). The network displayed many potential direct or indirect association. The network analysis suggested that the DENN domain containing the 4C (*DENND4C*) gene interacts with the hemoglobin subunit alpha 1 (*HBA1*) and HBS1-like translational GTPase (*HBS1L*) genes (22). The SUMO1/sentrin/SMT3-specific peptidase 2 (*SENP2*) gene was coexpressed with the egl-9 family hypoxia-inducible factor 1 (*EGLN1*) gene and was indirectly associated with many genes related to hematological traits via selenoprotein T (*SELENOT*) and small ubiquitin-like modifier 1 (*SUMO1*). Cathepsin B (*CTSB*) and oncostatin M receptor (*OSMR*) genes appeared to be directly associated with many genes related to hematological traits. The selectin P (*SELP*) gene showed shared protein domains and colocalization with the glycoprotein Ib platelet alpha subunit (*GP1BA*) gene. There was no information on association with the chromosome 8 open reading frame 37 (*C8orf37*) gene and HLA complex group 17 (*HCG17*) noncoding RNA in the analysis.

## DISCUSSION

In the longitudinal exome-wide association studies for 13 hematological traits, a total of 67 SNPs were significantly (FDR <0.01) related to hematological traits. Of the 67 SNPs, nine (rs4686683 of *SENP2* for RBCs; rs3133745 of *C8orf37-AS1* for WBCs; rs3917688 of *SELP* for MCV; rs13121954 at 4q31.2 for basophils; rs12338 of *CTSB* for neutrophils; rs395967 of *OSMR-AS1* for monocytes; and rs7584099 at 2q22.3, rs1579219 of *HCG17*, and rs10757049 of *DENND4C* for eosinophils) were identified as novel hematological markers (Supplemental Tables S1 and S2). The MAFs of the nine newly identified and 58 previously reported SNPs were 24–49% and 12–50%, respectively, and approxdf values of those SNPs were 33–223 and 35–647, respectively. Our longitudinal exome-wide association studies thus identified common variants related to hematological traits.

The GeneMANIA network suggested some potential direct or indirect relations between genes associated with hematological phenotypes and newly identified genes. In addition, some of the nine loci have plausible function. SENP2 protein is involved in the process of synthesized SUMO1 into conjugate form and activates to remove SUMO1 from its substrate. The SUMO protein plays an important role in essential biological function. This protein may cause hematological diseases such as acute myeloid leukemia (8, 47). Since the SENP2 protein is involved in the regulation of SUMOylation, the nucleotide substitution (C→A) at rs4686683 may affect the hematological dynamics. However, the candidate SNP is an intron variant. Therefore, the functional relevance of the candidate SNP to the hematological dynamics remains unclear.

The nonsynonymous substitution (G→C, L26V) at rs12338 in *CTSB* was significantly related to neutrophils in our longitudinal exome-wide association studies. *CTSB* is ubiquitously expressed in various tissues and organs, according to The Human Protein Atlas (http://www.proteinatlas.org/). This gene is a member of the C1 family of peptidases. The CTSB enzyme is released by neutrophils and cleaves the extracellular domain in CD18 molecules that play important roles in the regulation of neutrophil adhesion and migration (10, 39). Given that the CTSB enzyme is involved in neutrophil adhesion and migration through the CD18 cleavage, the association of *CTSB* with neutrophils may be attributable to the effect of this enzyme on the regulation in neutrophil dynamics.

The rs395967 (A→G) of *OSMR-AS1* showed significant association with monocytes. OSMR antisense RNA 1 (*OSMR-AS1*) is a noncoding RNA. *OSMR* is a member of interleukin (IL)-6 family cytokine receptors and is expressed in various tissues and organs (The Human Protein Atlas). Expression of *OSMR* and interleukin 31 receptor A (*IL31RA*) mRNA is induced in activated monocytes, and *OSMR* plays a role in inflammatory reactions and hematopoiesis (7, 42, 48). Given that *OSMR-AS1* regulates the expression of *OSMR*, the association of *OSMR-AS1* with monocytes might be attributable to the effect of this noncoding RNA on the regulation in monocyte dynamics.

The SELP protein is a 140 kDa adhesion molecule involved in the interaction of platelets with leukocytes. This protein is mainly distributed in megakaryocytes and platelets as well as vascular endothelial cells of diverse human organs (26). Knock-in mice with elevated plasma levels of soluble SELP showed several abnormalities related to atherosclerosis and cerebrovascular complications (17). In our longitudinal exome-wide association studies, rs3917688 (G→A) in *SELP* was significantly associated with MCV. However, this SNP was not associated with MCV in BCX studies (5, 9, 41) and Astle et al. (2016) (1) (*P* = 0.205–0.781). The minor allele “A” of rs3917688 in Asians (0.329–0.372) was major in European (0.509) and African populations (0.577), according to the 1000 Genomes Project. The difference of allele frequencies might be attributable to the discrepancy of association between SNPs and hematological phenotypes among ethnic groups. Although the effect direction of association in rs3917688 was same in the present study and the study of Astle et al. (2016), the effect of this SNP appeared to be stronger in Inabe subjects with East Asian ancestry (0.2748) than in subjects with European ancestry (0.0045). The functional relevance of SELP protein to MCV remains unclear, and further functional analysis is thus required to clarify the results of this study.

In the present study, SNPs in two noncoding RNA (*HCG17* and *C8orf37-AS1*) and one coding gene (*DENND4C*) were related to hematological traits. However, these noncoding RNA and gene have not been reported to show any association with hematological traits to date. Therefore, further functional analysis is required to verify the relations of these SNPs to hematological phenotypes. Of the nine SNPs newly identified in the present study, seven (rs7584099, rs3917688, rs13121954, rs12338, rs395967, rs1579219, and rs10757049) were detected from the GEE method in the recessive model only. It is possible that the recessive model may increase the chances of generating type I error due to potential genomic inflations. Therefore, replication studies are required to verify the association of the seven SNPs with hematological traits.

There are several limitations in the presents study. First, the longitudinal exome-wide association studies were conducted in a local Japanese population. Seven of the nine identified SNPs did not reach the threshold of *P* value with Bonferroni’s correction. In the examinations of WBC subtypes, the small sample size decreased the statistical power. Therefore, replication studies in other Japanese populations or other ethnic groups are required to verify the association of identified SNPs with hematological phenotypes. Second, the functional relevance of the nine SNPs identified in the present study to hematological traits remains unclear. Further functional analysis is required to clarify the results of this study. Third, genetic variants on sex chromosomes and mitochondrial DNA were not examined in our longitudinal exome-wide association studies. Forth, the follow-up period of annual health check-ups varied from 1 to 11 yr among individuals. An exome-wide association scan that tests the association of SNPs with time-dependent phenotypic changes is important. However, the time-dependent analysis with the R package bosswithdf does not allow any missing data. In future, a genome-wide time-dependent analysis is required to elucidate the association of SNPs with the time-varying phenotypes.

In conclusion, the GEE model showed that nine SNPs (rs4686683 of *SENP2*, rs3133745 of *C8orf37-AS1*, rs3917688 of *SELP*, rs13121954 at 4q31.2, rs12338 of *CTSB*, rs395967 of *OSMR-AS1*, rs7584099 at 2q22.3, rs1579219 of *HCG17*, and rs10757049 of *DENND4C*) were identified as novel hematological markers. The results of our longitudinal exome-wide association studies may contribute to the hematological study, and genotyping for these SNPs may be useful for precision medicine in Japanese.

## GRANTS

This work was supported by Research Grant from Okasan Kato Culture Promotion Foundation (to Y. Yasukochi), the Kurata Grant (no. 1323) awarded by the Hitachi Global Foundation (to Y. Yasukochi, Y. Yamada), CREST (JPMRJCR1302) of the Japan Science and Technology Agency (to Y. Yamada, J. Sakuma, I. Takeuchi), and by Japan Society for the Promotion of Science KAKENHI Grants JP17H00758 (to I. Takeuchi, Y. Yasukochi) and JP15H04772 (to Y. Yamada).

## DISCLOSURES

No conflicts of interest, financial or otherwise, are declared by the authors.

## AUTHOR CONTRIBUTIONS

Y. Yasukochi and Y. Yamada conceived and designed research; Y. Yasukochi, J.S., I.T., and Y. Yamada analyzed data; Y. Yasukochi, J.S., I.T., and Y. Yamada interpreted results of experiments; Y. Yasukochi prepared figures; Y. Yasukochi drafted manuscript; Y. Yasukochi, J.S., I.T., K.K., M.O., T.F., H.H., and Y. Yamada edited and revised manuscript; Y. Yasukochi, J.S., I.T., K.K., M.O., T.F., H.H., and Y. Yamada approved final version of manuscript; K.K., M.O., T.F., H.H., and Y. Yamada performed experiments.

## Supplemental Data

Tables S1-S4 Tables S1-S4 (.pdf 839 KB)

Table S5 Table S5 (.xlsx 55 KB)

Table S6 Table S6 (.xlsx 23 KB)

Table S7 Table S7 (.xlsx 77 KB)

Table S8 Table S8 (.xlsx 19 KB)

Table S9 Table S9 (.xlsx 23 KB)

Table S10 Table S10 (.xlsx 21 KB)
