# Supplementary material for: Identification of nine novel loci related to hematological traits in a Japanese population
Source: Physiol Genomics. 2018 Jun 29;50(9):758–69. doi: 10.1152/physiolgenomics.00088.2017 (PMC6172615; doi:10.1152/physiolgenomics.00088.2017)
Supplement: Tables S1-S4 — (.pdf 839 KB) [file TablesS1-S4.pdf]

Table S1. Candidate SNPs showing significant relations (FDR <0.01, approxdf >30) to hematological traits using the generalized estimating equation model with adjustments for age and gender.

Genetic model	Traits	RefSNP ID	Location[a]	Alleles (Major→ Minor)	Gene	Estimate[b]	*P*-value	FDR	MAF	Approxdf[c]
Additive	RBCs	rs218237	4: 54,528,005	C → T		-3.67	1.7×10^{-6}	0.003	0.289	47
		rs172629	4: 54,541,595	C → G		-3.79	8.3×10^{-7}	0.002	0.289	47
		rs11970772	6: 41,957,552	A → T	*CCND3*	3.83	1.9×10^{-7}	0.001	0.438	145
		rs11968166	6: 41,957,566	G → A	*CCND3*	-3.57	1.0×10^{-6}	0.002	0.449	141
		rs9376090	6: 135,090,090	T → C		-8.33	$< 2.0\times10^{-16}$	$< 4.9\times10^{-12}$	0.343	73
		rs7775698	6: 135,097,497	C → T		-8.35	$< 2.0\times10^{-16}$	$< 4.9\times10^{-12}$	0.341	73
		rs7776054	6: 135,097,778	A → G		-8.29	$< 2.0\times10^{-16}$	$< 4.9\times10^{-12}$	0.341	73
		rs9373124	6: 135,102,071	T → C		-6.64	$< 2.0\times10^{-16}$	$< 4.9\times10^{-12}$	0.374	101
		rs4895441	6: 135,105,435	A → G	*LOC105378010*	-6.74	$< 2.0\times10^{-16}$	$< 4.9\times10^{-12}$	0.377	104
		rs9376092	6: 135,106,006	C → A	*LOC105378010*	-6.71	$< 2.0\times10^{-16}$	$< 4.9\times10^{-12}$	0.377	104
		rs9402686	6: 135,106,679	G → A	*LOC105378010*	-6.71	$< 2.0\times10^{-16}$	$< 4.9\times10^{-12}$	0.377	104
		rs9494145	6: 135,111,414	T → C		-6.10	1.3×10^{-15}	1.6×10^{-11}	0.320	67
		rs9483788	6: 135,114,363	T → C		-5.80	1.2×10^{-14}	9.9×10^{-11}	0.343	81
		rs6569992	6: 135,131,014	G → A		-4.45	4.0×10^{-9}	2.5×10^{-5}	0.362	93
		rs8176749	9: 133,255,801	G → A	*ABO*	4.65	2.2×10^{-6}	0.003	0.160	76
		rs8176720	9: 133,257,486	A → G	*ABO*	3.51	1.5×10^{-6}	0.002	0.424	136
		rs651007	9: 133,278,431	G → A		-4.41	1.8×10^{-8}	7.4×10^{-5}	0.278	43
		rs579459	9: 133,278,724	T → C		-4.41	1.8×10^{-8}	7.4×10^{-5}	0.278	43
		rs635634	9: 133,279,427	G → A		-4.43	1.5×10^{-8}	7.4×10^{-5}	0.278	43
		rs507666	9: 136,149,399	G → A		-4.37	2.5×10^{-8}	8.8×10^{-5}	0.278	43
		rs6070697	20: 59,024,347	G → A	*TUBB1*	-5.18	1.8×10^{-6}	0.003	0.122	50
	Ht	rs9376090	6: 135,090,090	T → C		-0.35	9.3×10^{-8}	0.001	0.343	336
		rs7775698	6: 135,097,497	C → T		-0.36	5.6×10^{-8}	0.001	0.341	333
		rs7776054	6: 135,097,778	A → G		-0.35	8.1×10^{-8}	0.001	0.341	333
	MCV	rs172629	4: 54,541,595	C → G		0.66	2.2×10^{-10}	7.7×10^{-7}	0.289	248
		rs3218097	6: 41,937,537	C → T	*CCND3*	-0.56	4.1×10^{-6}	0.006	0.189	109

	rs9349205	6: 41,957,421	G → A	*CCND3*	-0.63	1.6×10^{-7}	3.6×10^{-4}	0.185	107
	rs9376090	6: 135,090,090	T → C		0.96	$< 2.0\times10^{-16}$	$< 4.9\times10^{-12}$	0.343	341
	rs7775698	6: 135,097,497	C → T		0.94	$< 2.0\times10^{-16}$	$< 4.9\times10^{-12}$	0.341	337
	rs7776054	6: 135,097,778	A → G		0.94	$< 2.0\times10^{-16}$	$< 4.9\times10^{-12}$	0.341	338
	rs9373124	6: 135,102,071	T → C		0.76	2.7×10^{-14}	1.7×10^{-10}	0.374	399
	rs4895441	6: 135,105,435	A → G	*LOC105378010*	0.77	1.3×10^{-14}	1.1×10^{-10}	0.377	404
	rs9376092	6: 135,106,006	C → A	*LOC105378010*	0.77	1.2×10^{-14}	1.5×10^{-10}	0.377	404
	rs9402686	6: 135,106,679	G → A	*LOC105378010*	0.77	1.2×10^{-14}	1.5×10^{-10}	0.377	404
	rs9494145	6: 135,111,414	T → C		0.76	7.0×10^{-14}	3.4×10^{-10}	0.320	302
	rs9483788	6: 135,114,363	T → C		0.70	2.3×10^{-12}	9.4×10^{-9}	0.343	342
	rs6569992	6: 135,131,014	G → A		0.58	3.5×10^{-9}	8.6×10^{-6}	0.362	376
	rs651007	9: 133,278,431	G → A		0.50	3.9×10^{-6}	0.006	0.278	41
	rs579459	9: 133,278,724	T → C		0.50	3.9×10^{-6}	0.006	0.278	41
	rs635634	9: 133,279,427	G → A		0.50	3.8×10^{-6}	0.006	0.278	41
	rs507666	9: 136,149,399	G → A		0.49	6.2×10^{-6}	0.008	0.278	41
	rs2235321	22: 37,066,886	G → A	*TMPRSS6*	0.46	1.4×10^{-6}	0.002	0.429	149
	rs855791	22: 37,066,896	T → C	*TMPRSS6*	0.57	3.2×10^{-9}	8.8×10^{-6}	0.425	480
	rs5756506	22: 37,071,352	G → C	*TMPRSS6*	0.47	5.8×10^{-7}	0.001	0.444	515
	rs4820268	22: 37,073,551	G → A	*TMPRSS6*	0.46	9.7×10^{-7}	0.002	0.444	514
	rs2413450	22: 37,074,184	T → C	*TMPRSS6*	0.47	7.4×10^{-7}	0.001	0.445	516
	rs140523	22: 50,524,353	C → G	*SCO2*	-0.50	8.0×10^{-6}	0.010	0.260	204
MCH	rs218237	4: 54,528,005	C → T		0.22	6.4×10^{-8}	1.8×10^{-4}	0.289	247
	rs172629	4: 54,541,595	C → G		0.23	2.9×10^{-8}	8.9×10^{-5}	0.289	248
	rs9376090	6: 135,090,090	T → C		0.39	$< 2.0\times10^{-16}$	$< 4.9\times10^{-12}$	0.343	341
	rs7775698	6: 135,097,497	C → T		0.38	$< 2.0\times10^{-16}$	$< 4.9\times10^{-12}$	0.341	337
	rs7776054	6: 135,097,778	A → G		0.38	$< 2.0\times10^{-16}$	$< 4.9\times10^{-12}$	0.341	338
	rs9373124	6: 135,102,071	T → C		0.31	1.3×10^{-15}	1.1×10^{-11}	0.374	399
	rs4895441	6: 135,105,435	A → G	*LOC105378010*	0.31	6.7×10^{-16}	8.3×10^{-12}	0.377	404
	rs9376092	6: 135,106,006	C → A	*LOC105378010*	0.31	6.7×10^{-16}	8.3×10^{-12}	0.377	404
	rs9402686	6: 135,106,679	G → A	*LOC105378010*	0.31	6.7×10^{-16}	8.3×10^{-12}	0.377	404

	rs9494145	6: 135,111,414	T → C		0.31	2.4×10^{-15}	1.5×10^{-11}	0.320	302
	rs9483788	6: 135,114,363	T → C		0.28	4.0×10^{-13}	2.0×10^{-9}	0.343	342
	rs6569992	6: 135,131,014	G → A		0.24	2.5×10^{-10}	8.8×10^{-7}	0.362	376
	rs2235321	22: 37,066,886	G → A	*TMPRSS6*	0.19	2.3×10^{-7}	0.001	0.429	490
	rs855791	22: 37,066,896	T → C	*TMPRSS6*	0.24	1.8×10^{-10}	7.4×10^{-7}	0.425	480
	rs5756506	22: 37,071,352	G → C	*TMPRSS6*	0.19	1.9×10^{-7}	4.7×10^{-4}	0.444	515
	rs4820268	22: 37,073,551	G → A	*TMPRSS6*	0.19	3.2×10^{-7}	0.001	0.444	514
	rs2413450	22: 37,074,184	T → C	*TMPRSS6*	0.19	1.9×10^{-7}	4.7×10^{-4}	0.445	515
MCHC	rs9376090	6: 135,090,090	T → C		0.07	1.9×10^{-9}	2.3×10^{-5}	0.343	392
	rs7775698	6: 135,097,497	C → T		0.07	1.8×10^{-9}	4.4×10^{-5}	0.341	336
	rs7776054	6: 135,097,778	A → G		0.07	2.6×10^{-9}	2.1×10^{-5}	0.341	333
	rs9373124	6: 135,102,071	T → C		0.06	2.0×10^{-7}	0.001	0.374	332
	rs4895441	6: 135,105,435	A → G	*LOC105378010*	0.06	1.8×10^{-7}	0.001	0.377	398
	rs9376092	6: 135,106,006	C → A	*LOC105378010*	0.06	2.2×10^{-7}	0.001	0.377	398
	rs9402686	6: 135,106,679	G → A	*LOC105378010*	0.06	2.2×10^{-7}	0.001	0.377	398
	rs9494145	6: 135,111,414	T → C		0.06	3.0×10^{-7}	0.001	0.320	297
	rs4737009	8: 41,772,887	G → A	*ANK1*	-0.06	3.3×10^{-7}	0.001	0.464	569
	rs3782886	12: 111,672,685	A → G	*BRAP*	-0.06	2.2×10^{-7}	0.001	0.318	291
	rs11066015	12: 111,730,205	G → A	*ACAD10*	-0.07	5.6×10^{-9}	3.4×10^{-5}	0.300	263
	rs671	12: 111,803,962	G → A	*ALDH2*	-0.07	9.0×10^{-9}	3.7×10^{-5}	0.301	263
	rs2074356	12: 112,207,597	C → T	*HECTD4*	-0.07	7.5×10^{-9}	3.7×10^{-5}	0.278	227
	rs11066280	12: 112,379,979	T → A	*HECTD4*	-0.06	1.1×10^{-7}	3.9×10^{-4}	0.313	284
	rs855791	22: 37,066,896	T → C	*TMPRSS6*	0.05	4.1×10^{-6}	0.008	0.425	138
Platelets	rs9376090	6: 135,090,090	T → C		0.63	2.1×10^{-8}	1.7×10^{-4}	0.343	72
	rs7775698	6: 135,097,497	C → T		0.64	1.1×10^{-8}	1.4×10^{-4}	0.341	72
	rs7776054	6: 135,097,778	A → G		0.65	8.8×10^{-9}	2.2×10^{-4}	0.341	72
WBCs	rs3094216	6: 31,116,271	T → C	*CDSN*	0.17	3.8×10^{-6}	0.006	0.227	144
	rs3130982	6: 31,116,298	C → G	*CDSN*	0.16	4.0×10^{-7}	0.003	0.353	340
	rs3094212	6: 31,117,993	T → C	*CDSN*	0.16	5.1×10^{-7}	0.002	0.353	340
	rs2240064	6: 31,146,796	T → C	*CCHCR1*	0.14	7.3×10^{-7}	0.002	0.461	525

		rs3131012	6: 31,147,664	C → T	*CCHCR1*	0.14	7.3×10^{-7}	0.002	0.461	525
		rs2073721	6: 31,161,839	G → A	*TCF19*	0.15	4.7×10^{-7}	0.002	0.452	506
		rs2073723	6: 31,162,301	C → T	*TCF19*	0.15	4.7×10^{-7}	0.002	0.452	506
		rs1065461	6: 31,162,725	G → A	*TCF19*	0.15	4.4×10^{-7}	0.003	0.452	506
		rs1419881	6: 31,162,816	C → T	*TCF19*	-0.14	3.0×10^{-6}	0.005	0.500	647
		rs3130931	6: 31,167,111	G → A	*POU5F1*	0.14	7.1×10^{-7}	0.002	0.455	510
		rs3130501	6: 31,168,676	G → A	*POU5F1*	0.14	7.6×10^{-7}	0.002	0.451	505
		rs3130502	6: 31,168,889	G → A	*POU5F1*	0.15	4.7×10^{-7}	0.002	0.452	506
		rs3094188	6: 31,174,468	T → G	*PSORS1C3*	0.16	2.2×10^{-6}	0.004	0.287	232
		rs3869115	6: 31,236,917	G → C		0.17	1.5×10^{-6}	0.003	0.225	144
		rs2844623	6: 31,264,766	G → A		0.17	8.4×10^{-7}	0.002	0.241	165
		rs2239707	6: 31,557,542	A → G	*NFKBIL1*	0.15	5.6×10^{-7}	0.002	0.389	417
		rs3133745	8: 95,522,578	C → T	*C8orf37-AS1*	-0.16	1.3×10^{-6}	0.003	0.239	163
		rs56030650	17: 39,974,934	A → C	*GSDMA*	-0.18	5.2×10^{-9}	6.4×10^{-5}	0.473	563
		rs4794822	17: 40,000,459	T → C		-0.18	3.2×10^{-9}	7.9×10^{-5}	0.490	595
	Monocytes	rs10107630	8: 129,591,389	T → C	*CCDC26*	0.38	2.5×10^{-7}	0.006	0.462	35
Dominant	RBCs	rs4686683	3: 185,589,575	C → A	*SENP2*	-4.85	5.5×10^{-6}	0.008	0.399	223
		rs11970772	6: 41,957,552	A → T	*CCND3*	5.68	5.1×10^{-7}	0.001	0.438	223
		rs9376090	6: 135,090,090	T → C		-8.68	$< 2.0\times10^{-16}$	$< 1.6\times10^{-12}$	0.343	243
		rs7775698	6: 135,097,497	C → T		-8.68	$< 2.0\times10^{-16}$	$< 4.9\times10^{-12}$	0.341	243
		rs7776054	6: 135,097,778	A → G		-8.58	$< 2.0\times10^{-16}$	$< 2.5\times10^{-12}$	0.341	243
		rs9373124	6: 135,102,071	T → C		-7.52	7.0×10^{-13}	2.9×10^{-9}	0.374	239
		rs4895441	6: 135,105,435	A → G	*LOC105378010*	-7.67	2.7×10^{-13}	1.7×10^{-9}	0.377	239
		rs9376092	6: 135,106,006	C → A	*LOC105378010*	-7.60	4.6×10^{-13}	2.3×10^{-9}	0.377	239
		rs9402686	6: 135,106,679	G → A	*LOC105378010*	-7.60	4.6×10^{-13}	2.3×10^{-9}	0.377	239
		rs9494145	6: 135,111,414	T → C		-6.67	7.9×10^{-11}	2.8×10^{-7}	0.320	249
		rs9483788	6: 135,114,363	T → C		-6.31	1.0×10^{-9}	3.1×10^{-6}	0.343	247
		rs8176746	9: 133,255,935	C → A	*ABO*	5.42	1.3×10^{-6}	0.002	0.169	84
		rs8176741	9: 133,256,074	C → T	*ABO*	5.39	1.3×10^{-6}	0.002	0.172	86
		rs7853989	9: 133,256,205	G → C	*ABO*	5.31	1.9×10^{-6}	0.003	0.172	86

	rs1053878	9: 133,256,264	C → T	*ABO*	-5.32	3.3×10^{-7}	0.001	0.228	159
	rs651007	9: 133,278,431	G → A		-5.62	3.8×10^{-8}	8.5×10^{-5}	0.278	228
	rs579459	9: 133,278,724	T → C		-5.62	3.8×10^{-8}	8.5×10^{-5}	0.278	228
	rs635634	9: 133,279,427	G → A		-5.67	3.0×10^{-8}	8.2×10^{-5}	0.278	228
	rs507666	9: 136,149,399	G → A		-5.63	3.6×10^{-8}	8.9×10^{-5}	0.278	228
	rs6070697	20: 59,024,347	G → A	*TUBB1*	-5.63	3.2×10^{-6}	0.005	0.122	50
MCV	rs218237	4: 54,528,005	C → T		0.78	1.7×10^{-8}	3.8×10^{-5}	0.289	247
	rs172629	4: 54,541,595	C → G		0.81	5.9×10^{-9}	1.5×10^{-5}	0.289	248
	rs9349205	6: 41,957,421	G → A	*CCND3*	-0.67	2.8×10^{-6}	0.004	0.185	107
	rs9376090	6: 135,090,090	T → C		1.07	1.7×10^{-14}	4.2×10^{-10}	0.343	254
	rs7775698	6: 135,097,497	C → T		1.04	7.9×10^{-14}	9.7×10^{-10}	0.341	254
	rs7776054	6: 135,097,778	A → G		1.04	9.5×10^{-14}	7.8×10^{-10}	0.341	254
	rs9373124	6: 135,102,071	T → C		0.92	1.1×10^{-10}	4.5×10^{-7}	0.374	251
	rs4895441	6: 135,105,435	A → G	*LOC105378010*	0.91	1.5×10^{-10}	4.6×10^{-7}	0.377	250
	rs9376092	6: 135,106,006	C → A	*LOC105378010*	0.92	1.3×10^{-10}	4.6×10^{-7}	0.377	250
	rs9402686	6: 135,106,679	G → A	*LOC105378010*	0.92	1.3×10^{-10}	4.6×10^{-7}	0.377	250
	rs9494145	6: 135,111,414	T → C		0.93	2.2×10^{-11}	1.4×10^{-7}	0.320	254
	rs9483788	6: 135,114,363	T → C		0.86	8.7×10^{-10}	2.4×10^{-6}	0.343	253
	rs6569992	6: 135,131,014	G → A		0.73	2.5×10^{-7}	4.1×10^{-4}	0.362	252
	rs2235321	22: 37,066,886	G → A	*TMPRSS6*	0.75	4.5×10^{-7}	0.001	0.429	254
	rs855791	22: 37,066,896	T → C	*TMPRSS6*	0.97	7.1×10^{-11}	3.5×10^{-7}	0.425	258
	rs5756506	22: 37,071,352	G → C	*TMPRSS6*	0.79	1.1×10^{-7}	2.3×10^{-4}	0.444	256
	rs4820268	22: 37,073,551	G → A	*TMPRSS6*	0.77	2.0×10^{-7}	3.5×10^{-4}	0.444	256
	rs2413450	22: 37,074,184	T → C	*TMPRSS6*	0.78	1.5×10^{-7}	2.8×10^{-4}	0.445	257
MCH	rs218237	4: 54,528,005	C → T		0.26	1.1×10^{-6}	0.002	0.289	247
	rs172629	4: 54,541,595	C → G		0.27	4.2×10^{-7}	0.001	0.289	248
	rs9376090	6: 135,090,090	T → C		0.44	7.8×10^{-16}	1.9×10^{-11}	0.343	254
	rs7775698	6: 135,097,497	C → T		0.43	4.6×10^{-15}	5.7×10^{-11}	0.341	254
	rs7776054	6: 135,097,778	A → G		0.42	6.6×10^{-15}	5.4×10^{-11}	0.341	254
	rs9373124	6: 135,102,071	T → C		0.37	2.6×10^{-11}	1.1×10^{-7}	0.374	250

	rs4895441	6: 135,105,435	A → G	*LOC105378010*	0.37	3.5×10^{-11}	1.1×10^{-7}	0.377	250
	rs9376092	6: 135,106,006	C → A	*LOC105378010*	0.37	2.8×10^{-11}	9.9×10^{-8}	0.377	250
	rs9402686	6: 135,106,679	G → A	*LOC105378010*	0.37	2.8×10^{-11}	9.9×10^{-8}	0.377	250
	rs9494145	6: 135,111,414	T → C		0.38	2.4×10^{-12}	1.5×10^{-8}	0.320	254
	rs9483788	6: 135,114,363	T → C		0.34	3.4×10^{-10}	9.3×10^{-7}	0.343	253
	rs6569992	6: 135,131,014	G → A		0.30	5.8×10^{-8}	1.4×10^{-4}	0.362	252
	rs2235321	22: 37,066,886	G → A	*TMPRSS6*	0.30	1.9×10^{-7}	3.3×10^{-4}	0.429	254
	rs855791	22: 37,066,896	T → C	*TMPRSS6*	0.39	1.7×10^{-11}	8.4×10^{-8}	0.425	258
	rs5756506	22: 37,071,352	G → C	*TMPRSS6*	0.32	6.7×10^{-8}	1.5×10^{-4}	0.444	256
	rs4820268	22: 37,073,551	G → A	*TMPRSS6*	0.31	1.3×10^{-7}	2.5×10^{-4}	0.444	256
	rs2413450	22: 37,074,184	T → C	*TMPRSS6*	0.31	7.6×10^{-8}	1.6×10^{-4}	0.445	257
MCHC	rs9376090	6: 135,090,090	T → C		0.08	1.1×10^{-7}	4.5×10^{-4}	0.343	250
	rs7775698	6: 135,097,497	C → T		0.08	2.6×10^{-7}	0.001	0.341	250
	rs7776054	6: 135,097,778	A → G		0.08	4.5×10^{-7}	0.001	0.341	250
	rs4737009	8: 41,772,887	G → A	*ANK1*	-0.08	2.1×10^{-6}	0.006	0.464	224
	rs3782886	12: 111,672,685	A → G	*BRAP*	-0.09	4.1×10^{-8}	2.0×10^{-4}	0.318	252
	rs11066015	12: 111,730,205	G → A	*ACAD10*	-0.09	3.5×10^{-9}	8.6×10^{-5}	0.300	250
	rs671	12: 111,803,962	G → A	*ALDH2*	-0.09	4.1×10^{-9}	5.1×10^{-5}	0.301	251
	rs2074356	12: 112,207,597	C → T	*HECTD4*	-0.09	2.4×10^{-8}	1.5×10^{-4}	0.278	227
	rs11066280	12: 112,379,979	T → A	*HECTD4*	-0.09	1.7×10^{-8}	1.4×10^{-4}	0.313	251
Platelets	rs9376090	6: 135,090,090	T → C		0.79	2.6×10^{-7}	0.003	0.343	241
	rs7775698	6: 135,097,497	C → T		0.79	2.1×10^{-7}	0.005	0.341	242
	rs7776054	6: 135,097,778	A → G		0.79	2.1×10^{-7}	0.005	0.341	242
WBCs	rs3094216	6: 31,116,271	T → C	*CDSN*	0.20	3.1×10^{-6}	0.010	0.227	144
	rs2240064	6: 31,146,796	T → C	*CCHCR1*	0.22	2.2×10^{-6}	0.009	0.461	245
	rs3131012	6: 31,147,664	C → T	*CCHCR1*	0.22	2.2×10^{-6}	0.009	0.461	245
	rs2073721	6: 31,161,839	G → A	*TCF19*	0.21	2.4×10^{-6}	0.008	0.452	248
	rs2073723	6: 31,162,301	C → T	*TCF19*	0.21	2.4×10^{-6}	0.008	0.452	248
	rs1065461	6: 31,162,725	G → A	*TCF19*	0.21	3.1×10^{-6}	0.010	0.452	248
	rs3130502	6: 31,168,889	G → A	*POU5F1*	0.21	2.4×10^{-6}	0.008	0.452	248

		rs3869115	6: 31,236,917	G → C		0.20	2.4×10^{-6}	0.008	0.225	144
		rs2844623	6: 31,264,766	G → A		0.21	1.2×10^{-6}	0.007	0.241	165
		rs2239707	6: 31,557,542	A → G	*NFKBIL1*	0.22	2.6×10^{-7}	0.002	0.389	233
		rs56030650	17: 39,974,934	A → C	*GSDMA*	-0.28	1.3×10^{-8}	3.2×10^{-4}	0.473	226
		rs4794822	17: 40,000,459	T → C		-0.28	2.8×10^{-8}	3.4×10^{-4}	0.490	223
Recessive	RBCs	rs11968166	6: 41,957,566	G → A	*CCND3*	-6.48	6.9×10^{-7}	0.002	0.449	141
		rs9376090	6: 135,090,090	T → C		-15.67	$< 2.0\times10^{-16}$	$< 4.9\times10^{-12}$	0.343	73
		rs7775698	6: 135,097,497	C → T		-15.78	$< 2.0\times10^{-16}$	$< 4.9\times10^{-12}$	0.341	73
		rs7776054	6: 135,097,778	A → G		-15.77	$< 2.0\times10^{-16}$	$< 4.9\times10^{-12}$	0.341	73
		rs9373124	6: 135,102,071	T → C		-10.97	1.9×10^{-13}	1.2×10^{-9}	0.374	101
		rs4895441	6: 135,105,435	A → G	*LOC105378010*	-11.06	8.6×10^{-14}	7.1×10^{-10}	0.377	104
		rs9376092	6: 135,106,006	C → A	*LOC105378010*	-11.09	7.2×10^{-14}	8.9×10^{-10}	0.377	104
		rs9402686	6: 135,106,679	G → A	*LOC105378010*	-11.09	7.2×10^{-14}	8.9×10^{-10}	0.377	104
		rs9494145	6: 135,111,414	T → C		-11.01	1.7×10^{-11}	8.4×10^{-8}	0.320	67
		rs9483788	6: 135,114,363	T → C		-10.39	1.7×10^{-11}	8.4×10^{-8}	0.343	81
		rs6569992	6: 135,131,014	G → A		-8.38	3.8×10^{-8}	1.6×10^{-4}	0.362	93
	Ht	rs9376090	6: 135,090,090	T → C		-0.77	3.0×10^{-8}	1.8×10^{-4}	0.343	72
		rs7775698	6: 135,097,497	C → T		-0.77	2.4×10^{-8}	2.0×10^{-4}	0.341	73
		rs7776054	6: 135,097,778	A → G		-0.77	2.2×10^{-8}	2.7×10^{-4}	0.341	73
	MCV	rs3917688	1: 169,621,842	G → A	*SELP*	1.05	2.4×10^{-6}	0.005	0.289	45
		rs9376090	6: 135,090,090	T → C		1.63	1.3×10^{-14}	3.2×10^{-10}	0.343	74
		rs7775698	6: 135,097,497	C → T		1.64	1.4×10^{-14}	1.7×10^{-10}	0.341	74
		rs7776054	6: 135,097,778	A → G		1.63	1.5×10^{-14}	1.2×10^{-10}	0.341	74
		rs9373124	6: 135,102,071	T → C		1.14	4.4×10^{-9}	1.5×10^{-5}	0.374	106
		rs4895441	6: 135,105,435	A → G	*LOC105378010*	1.18	8.5×10^{-10}	5.2×10^{-6}	0.377	109
		rs9376092	6: 135,106,006	C → A	*LOC105378010*	1.17	9.0×10^{-10}	4.4×10^{-6}	0.377	109
		rs9402686	6: 135,106,679	G → A	*LOC105378010*	1.17	9.0×10^{-10}	4.4×10^{-6}	0.377	109
		rs9494145	6: 135,111,414	T → C		1.11	2.0×10^{-7}	0.001	0.320	70
		rs9483788	6: 135,114,363	T → C		1.01	3.4×10^{-7}	0.001	0.343	84
	MCH	rs9376090	6: 135,090,090	T → C		0.64	4.7×10^{-15}	2.9×10^{-11}	0.343	74

	rs7775698	6: 135,097,497	C → T		0.65	2.2×10^{-15}	1.8×10^{-11}	0.341	74
	rs7776054	6: 135,097,778	A → G		0.65	2.1×10^{-15}	2.6×10^{-11}	0.341	74
	rs9373124	6: 135,102,071	T → C		0.47	3.0×10^{-10}	8.2×10^{-7}	0.374	106
	rs4895441	6: 135,105,435	A → G	*LOC105378010*	0.48	6.7×10^{-11}	2.4×10^{-7}	0.377	109
	rs9376092	6: 135,106,006	C → A	*LOC105378010*	0.48	7.9×10^{-11}	2.4×10^{-7}	0.377	109
	rs9402686	6: 135,106,679	G → A	*LOC105378010*	0.48	7.9×10^{-11}	2.4×10^{-7}	0.377	109
	rs9494145	6: 135,111,414	T → C		0.46	2.3×10^{-8}	5.7×10^{-5}	0.320	70
	rs9483788	6: 135,114,363	T → C		0.41	8.7×10^{-8}	1.9×10^{-4}	0.343	84
	rs6569992	6: 135,131,014	G → A		0.35	2.4×10^{-6}	0.005	0.362	96
Neutrophils	rs12338	8: 11,853,379	G → C	*CTSB*	-2.58	2.9×10^{-5}	0.007	0.495	49
Basophils	rs13121954	4: 147,102,677	G → A		-0.08	3.1×10^{-5}	0.007	0.493	47
Eosinophils	rs7584099	2: 147,720,767	G → A		-0.71	8.8×10^{-8}	0.000	0.427	33
	rs1579219	6: 30,256,528	G → A	*HCG17*	-0.65	2.0×10^{-5}	0.003	0.469	37
	rs10757049	9: 19,281,503	A → G	*DENND4C*	-0.58	5.6×10^{-5}	0.008	0.459	40
Monocytes	rs395967	5: 38,842,857	A → G	*OSMR-AS1*	-0.48	3.2×10^{-5}	0.008	0.482	49

[a] Location in NCBI build GRCh38. [b] Estimate of coefficient. [c] A scale of small effective sample size: approxdf = 2×MAF×Nindep, where Nindep is the sum of the estimated number of independent observations per person. SNP, single nucleotide polymorphism. RBC, red blood cell count. Ht, hematocrit. MCV, mean corpuscular volume. MCH, mean corpuscular hemoglobin. MCHC, mean corpuscular hemoglobin concentration. WBC, white blood cell count. FDR, false discovery rate. MAF, minor allele frequency.

Table S2. Significant SNPs identified by the GEE model for 13 hematological traits in Inabe residents.

RefSNP ID	Location[a]	Alleles	Gene	RBCs	Hb	Ht	MCV	MCH	MCHC	Plt	WBCs	Baso	Eosino	Neutro	Lympho	Mono
**Novel association**																
rs3917688	1: 169,621,842	G→A	*SELP*				✓									
rs7584099	2: 147,720,767	G→A											✓			
rs4686683	3: 185,589,575	C→A	*SENP2*	✓												
rs13121954	4: 147,102,677	G→A										✓				
rs395967	5: 38,842,857	A→G	*OSMR-AS1*													✓
rs1579219	6: 30,256,528	G→A	*HCG17*										✓			
rs12338	8: 11,853,379	G→C	*CTSB*											✓		
rs3133745	8: 95,522,578	C→T	*C8orf37-AS1*								✓					
rs10757049	9: 19,281,503	A→G	*DENND4C*										✓			
**Novel association (but possibly linked to known SNPs associated with any of hematological traits examined)**																
rs3094216	6: 31,116,271	T→C	*CDSN*								✓					
rs3130982	6: 31,116,298	C→G	*CDSN*								✓					
rs2240064	6: 31,146,796	T→C	*CCHCR1*								✓					
rs3131012	6: 31,147,664	C→T	*CCHCR1*								✓					
rs2073721	6: 31,161,839	G→A	*TCF19*								✓					
rs2073723	6: 31,162,301	C→T	*TCF19*								✓					
rs1065461	6: 31,162,725	G→A	*TCF19*								✓					
rs1419881	6: 31,162,816	C→T	*TCF19*								✓					
rs3130931	6: 31,167,111	G→A	*POU5F1*								✓					
rs3130501	6: 31,168,676	G→A	*POU5F1*								✓					
rs3130502	6: 31,168,889	G→A	*POU5F1*								✓					
rs3094188	6: 31,174,468	T→G	*PSORS1C3*								✓					
rs3869115	6: 31,236,917	G→C									✓					
rs2844623	6: 31,264,766	G→A									✓					
rs2239707	6: 31,557,542	A→G	*NFKBIL1*								✓					
rs10107630	8: 129,591,389	T→C	*CCDC26*													✓

rs8176741	9: 133,256,074	C→T	*ABO*	✓												
rs1053878	9: 133,256,264	C→T	*ABO*	✓												
rs635634	9: 133,279,427	G→A		✓			✓									
rs507666	9: 136,149,399	G→A		✓			✓									
rs3782886	12: 111,672,685	A→G	*BRAP*						✓							
rs11066015	12: 111,730,205	G→A	*ACAD10*						✓							
rs2074356	12: 112,207,597	C→T	*HECTD4*						✓							
rs11066280	12: 112,379,979	T→A	*HECTD4*						✓							
rs56030650	17: 39,974,934	A→C	*GSDMA*								✓					
**Replicated association (known SNPs associated with any of hematological traits examined)**																
rs218237	4: 54,528,005	C→T		✓			✓	✓								
rs172629	4: 54,541,595	C→G		✓			✓	✓								
rs3094212	6: 31,117,993	T→C	*CDSN*								✓					
rs3218097	6: 41,937,537	C→T	*CCND3*				✓									
rs9349205	6: 41,957,421	G→A	*CCND3*				✓									
rs11970772	6: 41,957,552	A→T	*CCND3*	✓												
rs11968166	6: 41,957,566	G→A	*CCND3*	✓												
rs9376090	6: 135,090,090	T→C		✓		✓	✓	✓	✓							
rs7775698	6: 135,097,497	C→T		✓		✓	✓	✓	✓	✓						
rs7776054	6: 135,097,778	A→G		✓		✓	✓	✓	✓	✓						
rs9373124	6: 135,102,071	T→C		✓			✓	✓	✓							
rs4895441	6: 135,105,435	A→G	*LOC105378010*	✓			✓	✓	✓							
rs9376092	6: 135,106,006	C→A	*LOC105378010*	✓			✓	✓	✓							
rs9402686	6: 135,106,679	G→A	*LOC105378010*	✓			✓	✓	✓							
rs9494145	6: 135,111,414	T→C		✓			✓	✓								
rs9483788	6: 135,114,363	T→C		✓			✓	✓								
rs6569992	6: 135,131,014	G→A		✓			✓	✓								
rs4737009	8: 41,772,887	G→A	*ANK1*						✓							
rs8176749	9: 133,255,801	G→A	*ABO*	✓												
rs8176746	9: 133,255,935	C→A	*ABO*	✓												

rs7853989	9: 133,256,205	G→C	*ABO*	✓												
rs8176720	9: 133,257,486	A→G	*ABO*	✓												
rs651007	9: 133,278,431	T→C		✓			✓									
rs579459	9: 133,278,724	G→A		✓			✓									
rs671	12: 111,803,962	G→A	*ALDH2*						✓							
rs4794822	17: 40,000,459	T→C									✓					
rs6070697	20: 59,024,347	G→A	*TUBB1*	✓												
rs2235321	22: 37,066,886	G→A	*TMPRSS6*				✓	✓								
rs855791	22: 37,066,896	T→C	*TMPRSS6*				✓	✓	✓							
rs5756506	22: 37,071,352	G→C	*TMPRSS6*				✓	✓								
rs4820268	22: 37,073,551	G→A	*TMPRSS6*				✓	✓								
rs2413450	22: 37,074,184	T→C	*TMPRSS6*				✓	✓								
rs140523	22: 50,524,353	C→G	*SCO2*				✓									
			**Total**	**26**	**0**	**3**	**25**	**17**	**14**	**2**	**19**	**1**	**3**	**1**	**0**	**2**

[a] Location in NCBI build GRCh38. SNP, single nucleotide polymorphism. GEE, generalized estimating equation. RBC, red blood cell count. Hb, hemoglobin. Ht, hematocrit. MCV, mean corpuscular volume. MCH, mean corpuscular hemoglobin. MCHC, mean corpuscular hemoglobin concentration. Plt, platelets. WBC, white blood cell count. Baso, basophils. Eosino, eosinophils. Neutro, neutrophils. Lympho, lymphocytes. Mono, monocytes. A tick represents a significant association between the corresponding SNP and hematological trait, according to a false discovery rate of <0.01 in the GEE model.

Table S3. Allele frequency of each candidate SNP in the Inabe cohort.

RefSNP ID	Location[a]	Gene	Frequency
rs3917688	1: 169,621,842	*SELP*	G: 0.711 (6946)
			A: 0.289 (2822)
rs7584099	2: 147,720,767		G: 0.573 (5593)
			A: 0.427 (4175)
rs4686683	3: 185,589,575	*SENP2*	C: 0.600 (5862)
			A: 0.400 (3906)
rs218237	4: 54,528,005		C: 0.711 (6946)
			T: 0.289 (2818)
rs172629	4: 54,541,595		C: 0.712 (6952)
			G: 0.288 (2816)
rs13121954	4: 147,102,677		G: 0.508 (4960)
			A: 0.492 (4808)
rs395967	5: 38,842,857	*OSMR-AS1*	A: 0.517 (5050)
			G: 0.483 (4718)
rs1579219	6: 30,256,528	*HCG17*	G: 0.532 (5198)
			A: 0.468 (4570)
rs3094216	6: 31,116,271	*CDSN*	T: 0.773 (7554)
			C: 0.227 (2214)
rs3130982	6: 31,116,298	*CDSN*	C: 0.648 (6330)
			G: 0.352 (3438)
rs3094212	6: 31,117,993	*CDSN*	T: 0.648 (6331)
			C: 0.352 (3437)
rs2240064	6: 31,146,796	*CCHCR1*	T: 0.538 (5252)
			C: 0.462 (4516)
rs3131012	6: 31,147,664	*CCHCR1*	C: 0.538 (5252)
			T: 0.462 (4516)
rs2073721	6: 31,161,839	*TCF19*	G: 0.547 (5347)
			A: 0.453 (4421)
rs2073723	6: 31,162,301	*TCF19*	C: 0.547 (5347)
			T: 0.453 (4421)
rs1065461	6: 31,162,725	*TCF19*	G: 0.547 (5341)
			A: 0.453 (4427)
rs1419881	6: 31,162,816	*TCF19*	C: 0.501 (4870)
			T: 0.499 (4850)
rs3130931	6: 31,167,111	*POU5F1*	G: 0.544 (5316)
			A: 0.456 (4452)
rs3130501	6: 31,168,676	*POU5F1*	G: 0.548 (5350)
			A: 0.452 (4418)
rs3130502	6: 31,168,889	*POU5F1*	G: 0.547 (5347)
			A: 0.453 (4421)
rs3094188	6: 31,174,468	*PSORS1C3*	T: 0.714 (6975)
			G: 0.286 (2793)
rs3869115	6: 31,236,917		G: 0.776 (7577)
			C: 0.224 (2191)
rs2844623	6: 31,264,766		G: 0.759 (7416)

			A: 0.241 (2350)
rs2239707	6: 31,557,542	*NFKBIL1*	A: 0.612 (5979)
			G: 0.388 (3789)
rs3218097	6: 41,937,537	*CCND3*	C: 0.810 (7912)
			T: 0.190 (1854)
rs9349205	6: 41,957,421	*CCND3*	G: 0.815 (7957)
			A: 0.185 (1811)
rs11970772	6: 41,957,552	*CCND3*	A: 0.558 (5450)
			T: 0.442 (4318)
rs11968166	6: 41,957,566	*CCND3*	G: 0.554 (5407)
			A: 0.446 (4361)
rs9376090	6: 135,090,090		T: 0.659 (6441)
			C: 0.341 (3327)
rs7775698	6: 135,097,497		C: 0.661 (6459)
			T: 0.339 (3309)
rs7776054	6: 135,097,778		A: 0.661 (6455)
			G: 0.339 (3313)
rs9373124	6: 135,102,071		T: 0.628 (6139)
			C: 0.372 (3629)
rs4895441	6: 135,105,435	*LOC105378010*	A: 0.626 (6117)
			G: 0.374 (3651)
rs9376092	6: 135,106,006	*LOC105378010*	C: 0.626 (6114)
			A: 0.374 (3654)
rs9402686	6: 135,106,679	*LOC105378010*	G: 0.626 (6114)
			A: 0.374 (3654)
rs9494145	6: 135,111,414		T: 0.682 (6661)
			C: 0.318 (3107)
rs9483788	6: 135,114,363		T: 0.659 (6435)
			C: 0.341 (3333)
rs6569992	6: 135,131,014		G: 0.641 (6261)
			A: 0.359 (3507)
rs12338	8: 11,853,379	*CTSB*	G: 0.506 (4938)
			C: 0.494 (4826)
rs4737009	8: 41,772,887	*ANK1*	G: 0.537 (5244)
			A: 0.463 (4524)
rs3133745	8: 95,522,578	*C8orf37-AS1*	C: 0.757 (7399)
			T: 0.243 (2369)
rs10107630	8: 129,591,389	*CCDC26*	T: 0.538 (5250)
			C: 0.462 (4514)
rs10757049	9: 19,281,503	*DENND4C*	A: 0.540 (5275)
			G: 0.460 (4491)
rs8176749	9: 133,255,801	*ABO*	G: 0.841 (8216)
			A: 0.159 (1550)
rs8176746	9: 133,255,935	*ABO*	C: 0.833 (8132)
			A: 0.167 (1636)
rs8176741	9: 133,256,074	*ABO*	C: 0.830 (8107)
			T: 0.170 (1661)
rs7853989	9: 133,256,205	*ABO*	G: 0.830 (8078)

			C: 0.170 (1656)
rs1053878	9: 133,256,264	*ABO*	C: 0.772 (7541)
			T: 0.228 (2227)
rs8176720	9: 133,257,486	*ABO*	A: 0.577 (5633)
			G: 0.423 (4135)
rs651007	9: 133,278,431		G: 0.722 (7051)
			A: 0.278 (2717)
rs579459	9: 133,278,724		T: 0.722 (7051)
			C: 0.278 (2717)
rs635634	9: 133,279,427		G: 0.722 (7053)
			A: 0.278 (2715)
rs507666	9: 136,149,399		G: 0.722 (7057)
			A: 0.278 (2711)
rs3782886	12: 111,672,685	*BRAP*	A: 0.682 (6666)
			G: 0.318 (3102)
rs11066015	12: 111,730,205	*ACAD10*	G: 0.699 (6832)
			A: 0.301 (2936)
rs671	12: 111,803,962	*ALDH2*	G: 0.699 (6829)
			A: 0.301 (2939)
rs2074356	12: 112,207,597	*HECTD4*	C: 0.723 (7060)
			T: 0.277 (2708)
rs11066280	12: 112,379,979	*HECTD4*	T: 0.686 (6701)
			A: 0.314 (3067)
rs56030650	17: 39,974,934	*GSDMA*	A: 0.526 (5134)
			C: 0.474 (4626)
rs4794822	17: 40,000,459		T: 0.510 (4982)
			C: 0.490 (4786)
rs6070697	20: 59,024,347	*TUBB1*	G: 0.880 (8597)
			A: 0.120 (1171)
rs2235321	22: 37,066,886	*TMPRSS6*	G: 0.570 (5572)
			A: 0.430 (4196)
rs855791	22: 37,066,896	*TMPRSS6*	T: 0.574 (5607)
			C: 0.426 (4161)
rs5756506	22: 37,071,352	*TMPRSS6*	G: 0.555 (5423)
			C: 0.445 (4345)
rs4820268	22: 37,073,551	*TMPRSS6*	G: 0.556 (5427)
			A: 0.444 (4341)
rs2413450	22: 37,074,184	*TMPRSS6*	T: 0.555 (5418)
			C: 0.445 (4350)
rs140523	22: 50,524,353	*SCO2*	C: 0.740 (7231)
			G: 0.260 (2535)

[a] Location in NCBI build GRCh38. SNP, single nucleotide polymorphism.

Table S4. Mean quantitative values of 13 hematological traits in Inabe subjects with different genotypes at nine SNPs identified by the GEE models.

Traits	RefSNP ID	Location[a]	Gene	Genotype	RBC	Hb	Ht	MCV	MCH	MCHC	Platelet	WBC	Neutrophil	Basophil	Eosinophil	Lymphocyte	Monocyte
**RBCs**	rs4686683	3: 185,589,575	*SENP2*	CC	**4.43±0.46**	13.93±1.60	40.97±4.54	92.51±5.60	31.45±2.19	33.98±0.72	235.03±57.50	5.63±1.73	57.87±8.76	0.66±0.46	3.12±2.17	31.22±8.01	7.13±2.05
				CA	**4.40±0.47**	13.86±1.60	40.76±4.52	92.70±5.56	31.52±2.16	33.99±0.72	232.92±58.27	5.50±1.64	57.25±9.54	0.69±0.48	3.54±2.65	31.28±8.65	7.20±2.11
				AA	**4.41±0.49**	13.78±1.68	40.56±4.72	92.16±5.77	31.29±2.27	33.93±0.74	233.97±57.37	5.52±1.67	58.28±9.50	0.69±0.46	3.47±2.67	30.30±8.36	7.23±1.99
**MCV**	rs3917688	1: 169,621,842	*SELP*	GG	4.42±0.47	13.86±1.60	40.77±4.52	**92.43±5.70**	31.41±2.24	33.97±0.73	233.16±56.94	5.56±1.70	57.25±9.43	0.69±0.48	3.43±2.48	31.39±8.60	7.22±2.09
				GA	4.42±0.48	13.89±1.62	40.86±4.58	**92.46±5.59**	31.43±2.17	33.98±0.72	234.61±59.24	5.53±1.66	58.12±8.94	0.66±0.46	3.24±2.45	30.77±7.95	7.18±2.07
				AA	4.36±0.49	13.87±1.68	40.77±4.74	**93.62±5.04**	31.85±1.99	34.00±0.72	234.51±56.33	5.56±1.65	57.49±9.85	0.67±0.45	3.80±2.78	31.08±9.16	6.94±1.93
**Neutrophil**	rs12338	8: 11,853,379	*CTSB*	GG	4.42±0.49	13.86±1.64	40.78±4.66	92.43±5.71	31.41±2.22	33.96±0.72	235.64±56.17	5.59±1.67	**57.69±9.00**	0.66±0.46	3.41±2.51	30.91±8.19	7.30±2.06
				GC	4.42±0.46	13.90±1.59	40.87±4.49	92.59±5.50	31.48±2.15	33.99±0.72	233.70±57.97	5.59±1.70	**58.37±8.95**	0.68±0.47	3.28±2.40	30.51±8.03	7.13±2.10
				CC	4.40±0.48	13.84±1.64	40.71±4.62	92.55±5.72	31.46±2.24	33.97±0.73	232.48±59.24	5.43±1.63	**55.79±10.06**	0.68±0.48	3.60±2.71	32.73±9.20	7.16±2.01
**WBCs**	rs3133745	8: 95,522,578	*C8orf37-AS1*	CC	4.42±0.47	13.87±1.60	40.80±4.53	92.49±5.46	31.44±2.13	33.97±0.71	234.27±58.20	**5.59±1.72**	57.97±9.11	0.68±0.47	3.33±2.44	30.84±8.28	7.16±2.00
				CT	4.42±0.47	13.89±1.64	40.84±4.62	92.59±5.93	31.48±2.33	33.98±0.75	234.33±58.13	**5.53±1.65**	57.03±9.48	0.68±0.47	3.46±2.63	31.54±8.55	7.25±2.19
				TT	4.39±0.49	13.83±1.60	40.67±4.51	92.76±5.08	31.54±1.98	33.99±0.71	227.68±52.52	**5.30±1.43**	57.73±9.51	0.66±0.47	3.42±2.39	31.15±8.43	6.99±1.99
**Basophil**	rs13121954	4: 147,102,677		GG	4.39±0.48	13.77±1.67	40.53±4.69	92.44±5.73	31.40±2.25	33.95±0.75	231.70±55.57	5.49±1.63	57.92±9.33	**0.69±0.46**	3.39±2.45	30.72±8.47	7.24±2.06
				GA	4.42±0.47	13.91±1.62	40.90±4.56	92.56±5.64	31.47±2.20	33.98±0.72	233.76±58.91	5.53±1.72	57.15±9.26	**0.70±0.49**	3.45±2.56	31.48±8.46	7.22±2.10
				AA	4.42±0.46	13.91±1.55	40.90±4.41	92.60±5.42	31.50±2.10	34.00±0.69	236.41±57.94	5.66±1.65	58.23±9.19	**0.62±0.44**	3.25±2.45	30.81±8.15	7.03±2.00
**Eosinophil**	rs7584099	2: 147,720,767		GG	4.44±0.47	13.97±1.63	41.07±4.61	92.53±5.57	31.47±2.19	33.99±0.73	232.47±56.32	5.53±1.70	57.16±9.13	0.67±0.47	**3.43±2.46**	31.43±8.23	7.29±2.15
				GA	4.40±0.47	13.81±1.60	40.63±4.52	92.48±5.62	31.43±2.19	33.97±0.72	234.71±57.10	5.56±1.66	57.60±9.21	0.69±0.48	**3.56±2.71**	30.99±8.33	7.12±2.04
				AA	4.41±0.48	13.87±1.62	40.80±4.56	92.71±5.65	31.52±2.19	33.98±0.73	234.15±62.32	5.56±1.71	58.60±9.65	0.65±0.45	**2.83±1.86**	30.78±8.82	7.12±1.99
	rs1579219	6: 30,256,528	*HCG17*	GG	4.41±0.47	13.87±1.62	40.78±4.57	92.58±5.59	31.48±2.19	33.98±0.73	231.66±55.31	5.50±1.65	57.79±9.20	0.67±0.47	**2.86±2.17**	30.82±8.42	7.14±2.01
				GA	4.42±0.48	13.90±1.62	40.87±4.59	92.61±5.55	31.49±2.16	33.98±0.72	234.72±58.83	5.56±1.66	57.38±9.52	0.68±0.48	**3.52±2.66**	31.18±8.50	7.22±2.11
				AA	4.41±0.46	13.83±1.60	40.69±4.49	92.32±5.77	31.37±2.26	33.96±0.74	234.81±58.73	5.60±1.76	58.01±8.78	0.68±0.45	**3.52±2.40**	31.31±8.07	7.12±2.06
	rs10757049	9: 19,281,503	*DENND4C*	AA	4.40±0.48	13.83±1.63	40.66±4.60	92.49±5.54	31.44±2.16	33.98±0.72	235.06±58.42	5.57±1.69	56.81±9.07	0.69±0.50	**3.58±2.77**	31.65±8.24	7.21±2.08
				AG	4.43±0.47	13.89±1.61	40.86±4.54	92.43±5.73	31.42±2.23	33.97±0.73	233.86±58.21	5.54±1.67	57.68±9.33	0.68±0.46	**3.43±2.50**	31.00±8.48	7.19±2.07
				GG	4.41±0.47	13.90±1.62	40.89±4.56	92.86±5.43	31.57±2.13	33.99±0.73	232.24±56.20	5.55±1.68	58.67±9.34	0.65±0.44	**2.97±2.01**	30.57±8.35	7.10±2.04
**Monocytes**	rs395967	5: 38,842,857	*OSMR-AS1*	AA	4.42±0.47	13.87±1.64	40.81±4.60	92.41±5.73	31.4±2.25	33.96±0.75	233.84±61.25	5.54±1.67	57.77±9.18	0.67±0.47	3.56±2.50	30.83±8.42	**7.16±1.98**
				AG	4.41±0.47	13.88±1.62	40.81±4.57	92.63±5.55	31.49±2.17	33.98±0.72	234.64±57.43	5.53±1.65	57.43±9.48	0.67±0.46	3.30±2.51	31.26±8.45	**7.32±2.14**
				GG	4.42±0.48	13.87±1.59	40.78±4.52	92.51±5.61	31.45±2.17	33.98±0.71	232.25±54.60	5.62±1.75	57.91±8.93	0.69±0.50	3.33±2.49	31.11±8.22	**6.86±1.99**

[a] Location in NCBI build GRCh38. Quantitative data are means and standard deviations. RBC, red blood cell count. Hb, hemoglobin. Ht, hematocrit. MCV, mean corpuscular volume. MCH, mean corpuscular hemoglobin. MCHC, mean corpuscular hemoglobin concentration. WBC, white blood cell count. Mean quantitative values of hematological traits related to each SNP are shown in bold.
